# Fatty Acids Derivatives From Eukaryotic Microalgae, Pathways and Potential Applications

**DOI:** 10.3389/fmicb.2021.718933

**Published:** 2021-09-29

**Authors:** Martina Blasio, Sergio Balzano

**Affiliations:** ^1^Department of Marine Biotechnologies, Stazione Zoologica Anton Dohrn Napoli (SZN), Naples, Italy; ^2^Department of Marine Microbiology and Biogeochemistry, Royal Netherlands Institute for Sea Research (NIOZ), Den Burg (Texel), Netherlands

**Keywords:** fatty acid derivatives, microalgal metabolites, secondary functional groups, biofuels, biopolymers

## Abstract

The exploitation of petrochemical hydrocarbons is compromising ecosystem and human health and biotechnological research is increasingly focusing on sustainable materials from plants and, to a lesser extent, microalgae. Fatty acid derivatives include, among others, oxylipins, hydroxy fatty acids, diols, alkenones, and wax esters. They can occur as storage lipids or cell wall components and possess, in some cases, striking cosmeceutical, pharmaceutical, and nutraceutical properties. In addition, long chain (>20) fatty acid derivatives mostly contain highly reduced methylenic carbons and exhibit a combustion enthalpy higher than that of C_14__–__20_ fatty acids, being potentially suitable as biofuel candidates. Finally, being the building blocks of cell wall components, some fatty acid derivatives might also be used as starters for the industrial synthesis of different polymers. Within this context, microalgae can be a promising source of fatty acid derivatives and, in contrast with terrestrial plants, do not require arable land neither clean water for their growth. Microalgal mass culturing for the extraction and the exploitation of fatty acid derivatives, along with products that are relevant in nutraceutics (e.g., polyunsaturated fatty acids), might contribute in increasing the viability of microalgal biotechnologies. This review explores fatty acids derivatives from microalgae with applications in the field of renewable energies, biomaterials and pharmaceuticals. *Nannochloropsis* spp. (Eustigmatophyceae, Heterokontophyta) are particularly interesting for biotechnological applications since they grow at faster rates than many other species and possess hydroxy fatty acids and aliphatic cell wall polymers.

## Microalgae as a Source of Specialty Compounds

Due to the dramatic environmental issues caused by the consumption of fossil fuels as well as other materials of petrochemical origin, the use of biological resources for the production of sustainable compounds as well as biofuels is receiving greater attention from the chemical industry. The increasing demand of sustainable materials led the scientific community to address their studies in the research of new molecules. Biotechnological research investigated terrestrial plants for several decades highlighting a number of potential applications for the pharmaceutical, cosmeceutical, and nutraceutical industries. For examples, extracts from plants such as *Simmondsia chinensis* (Pazyar et al., 2013), *Argania spinosa* (Avsar et al., 2016), and *Aloe vera* (Hekmatpou et al., 2019) are commonly used for skin protection and personal care products. A recent and exhaustive review on cosmeceutical and pharmaceutical products from plants is provided by Dorni et al. (2017).

Although natural products have been mostly isolated from plants, microalgal biodiversity represents an unexplored source of specialty molecules with potential biotechnological applications. A significant portion of marine diversity is unknown since most marine microbes are unculturable (Rappé and Giovannoni, 2003) and genomes or transcriptomes have been sequenced from a fraction of species only (Lewis et al., 2020). The number of sequenced genomes in eukaryotes is far lower compared to prokaryotes because the presence of both introns and tandem repeats significantly complicates eukaryotic genome sequencing (Richter et al., 2020); massive efforts have been carried out to sequence microalgal genomes (Blaby-Haas and Merchant, 2019) and transcriptomes (Keeling et al., 2014) from microbial eukaryotes and a database of proteins predicted from 742 eukaryotic genomes or transcriptomes has been recently published (Richter et al., 2020). Finally, a significant portion of the genes and transcripts from sequenced genomes and transcriptomes has not been investigated in great detail and their function is yet unknown. The great abundance of unannotated genes along with the scarcity of sequenced eukaryotic genomes suggest a great level of novelty potentially occurring within microalgal metabolites.

Microalgae appear to be more suitable than plants for mass culturing and specialty product development for a number of reasons. First, microalgae can achieve strikingly high growth rates reaching very short (1–2 days) doubling times (Chisti, 2007; Rodolfi et al., 2009; Stoytcheva and Montero, 2012). Conversely to crop plants that require cultivable lands and clean water, microalgae can grow in a range of different environments including brackish, marine or hypersaline waters as well as wastewaters and thus do not compete with food crops for arable land. Second, microalgae encompass a phylogenetic diversity much broader than that of terrestrial plants (Baldauf, 2008), potentially reflecting a greater diversity of metabolites, some of which might reveal useful for biotechnological purposes. It has been estimated that microalgae make up 200,000 to several million species worldwide versus the 250,000 species of higher plants (Norton et al., 1996; Mann and Vanormelingen, 2013). Microalgae produce a wide variety of fatty acid derivatives, such as hydroxy fatty acids, oxylipins, alkenones and diols, which can make an additional pool of molecules with potential biotechnological applications. The biochemical composition of microalgal biomass can be optimized (e.g., increase in lipids) according to the application requirement using appropriate culturing manipulations. A wide range of microalgal lipids are currently attracting some biotechnological interest. Polyunsaturated fatty acids (PUFAs) from microalgae have been widely investigated for nutraceutical applications (Doughman et al., 2007; Sathasivam et al., 2019) as well as sustainable alternatives to petroleum-based diesel (Nascimento et al., 2013). In addition to microalgae, thraustochytrids are a group of heterotrophic heterokonts that can also accumulate great amounts of PUFAs and are also under investigation because of their biotechnological potential (Fossier Marchan et al., 2018; Hu et al., 2020).

Biofuel combustion has far less emissions of sulfur compounds compared to petrochemical fuels (Khan et al., 2018). Furthermore, the biomass to be used for biofuel production has a great potential for fixing carbon dioxide from the air or from flue gas emissions. Oil extraction yields from microalgae can exceed by 5–6 fold that of terrestrial plants (Rodolfi et al., 2009; Mata et al., 2010; Benedetti et al., 2018). Finally, microalgae convert carbon dioxide in organic carbon with a greater efficiency than terrestrial plants (Melis, 2009; Zhu et al., 2010; Bhola et al., 2014). Microalgal species suitable for industrial applications need to perform fast growth, high lipid content, and minimum nutritional requests. Among all species, the green algal genera *Chlorella*, *Botryococcus*, and *Scenedesmus*, the diatom *Phaeodactylum tricornutum*, the Haptophyta *Isochrysis galbana* and Eustigmatophyceae from the genus *Nannochloropsis* can reach a high lipid content revealing suitable for biofuel development (Rodolfi et al., 2009; Shuba and Kifle, 2018). *Nannochloropsis* spp. have been long considered one of the most suitable candidates for biofuel production because of their fast growth and their high lipid content with respect to other species (Rodolfi et al., 2009; Chen et al., 2012). The content in triacylglycerols (TAGs) within selected strains of *Nannochloropsis oceanica* and *Nannochloropsis oculata* can indeed make up to 58% of total lipids (Ma et al., 2014). The potential of microalgae for biofuel development has been extensively discussed in a number of reviews (Chisti, 2007; Khan et al., 2018; Shuba and Kifle, 2018) and is beyond the scope of the present study. In this review we focus on the applications of fatty acid derivatives for a number of applications including biofuels and fuel additives.

## Microalgal Fatty Acids

### Fatty Acid Diversity

Fatty acids typically contain 14 to 24 carbon atoms and can be saturated, monounsaturated (MUFAs), or PUFAs. The most common saturated fatty acids in nature are the C_16__:__0_ (palmitic) and C_18__:__0_ (stearic) fatty acids and, to a lesser extent, the C_12__:__0_, C_14__:__0_, and C_20__:__0_ fatty acids. C_14__–__20_ saturated fatty acids are the major components of both storage and membrane lipids. Very long chain (>20) saturated fatty acids (VLCFAs) are common in terrestrial plants and can be the building blocks of plant waxes (Samuels et al., 2008). MUFAs are instead mostly represented by C_18__:__1_ω9 (oleic acid) as well as C_16__:__1_ω7, C_18__:__1_ω7, C_20__:1_ω11, and C_22__:__1_ω9; they are particularly abundant in avocados, olives, and nuts. PUFAs are abundant in phospholipid fatty acids being thus essential components of cell membranes. The most abundant PUFAs in plants are the C_18__:__2_ω6 (linoleic acid), the C_18__:__3_ω3 (α-linolenic acid), and the C_18__:__3_ω6 (γ-linolenic acid) that are mostly present in seeds and nuts. In contrast, protists possess longer PUFAs, the most common being the C_20__:__5_ω3 (eicosapentaenoic acid, EPA), the C_22__:__6_ω3 (docosahexaenoic acid, DHA), and the and C_20__:__4_ω6 (arachidonic acid, ARA).

PUFAs, especially ω3-PUFAs, are strongly recommended for human diet because they exert positive effects on health reducing the risks of cardiovascular diseases (Briggs et al., 2017), atherosclerosis (Dessì et al., 2013) as well as cancer and inflammation diseases (Gu et al., 2015). Furthermore, ω6-PUFAs such as γ-linolenic acid and ARA are also recommended, in small amounts, for human and animal diets since they can be precursors of ω3-PUFAs (Meyer et al., 2003). Although all organisms are able to biosynthesise PUFAs, the conversion efficiency in humans and other animals is not sufficient to fulfill the nutritional needs. PUFAs can be supplied, at a limited extent, by vascular plants that are rich in linoleic and α-linolenic acids, but produce very small amounts of EPA and DHA (Robert et al., 2005). EPA and DHA are mostly produced by microalgae and transferred to the upper levels of the food chain. At present, the major commercial sources of PUFAs are fish oils, which are mostly produced from wild-caught fishes, rising concerns for the sustainability of fishing stocks and the depletion of marine resources. Subsequently, great effort is being made to find alternative strategies for PUFA production. The microalgal content in EPA and DHA is far larger than that of plants. In addition some species also contain substantial proportions of typical plant PUFAs such as C_16__:__4_ω3 acid, α- and γ-linolenic acids, and C_18__:__4_ω3 acid (Sathasivam et al., 2019) and are thus attracting the attention of the biotechnological industry (Table 1).

**TABLE 1:** Major producers of polyunsaturated fatty acids (PUFAs).

Major producing genera	Class	Compound of interest	References
*Ankistrodesmus*	Chlorophyta	Hexadecatetraenoic acid (C_16__:__4_ω3)	Sathasivam et al., 2019
*Chlamydomonas*	Chlorophyta	Hexadecatetraenoic acid (C_16__:__4_ω3)	Ramanan et al., 2013
*Dunaliella*	Chlorophyta	Hexadecatetraenoic acid (C_16__:__4_ω3)	Lee et al., 2014
*Tetraselmis*	Chlorophyta	Hexadecatetraenoic acid (C_16__:__4_ω3)	Shin et al., 2018
*Botryococcus*	Chlorophyta	Linoleic acid (C_18__:__2_ω6)	Pérez-Mora et al., 2016
Chlorella	Chlorophyta	Linoleic acid (C_18__:__2_ω6)	Kholif et al., 2017
*Chlorococcum*	Chlorophyta	Linoleic acid (C_18__:__2_ω6)	Bhagavathy et al., 2011
*Tetraselmis*	Chlorophyta	Linoleic acid (C_18__:__2_ω6)	Kim et al., 2016
*Botryococcus*	Chlorophyta	α-linolenic acid (C_18__:__3_ω3)	Pérez-Mora et al., 2016
*Chlamydomonas*	Chlorophyta	α-linolenic acid (C_18__:__3_ω3)	Ramanan et al., 2013
*Chlorella*	Chlorophyta	α-linolenic acid (C_18__:__3_ω3)	Kholif et al., 2017
*Dunaliella*	Chlorophyta	α-linolenic acid (C_18__:__3_ω3)	Chen et al., 2015
*Micromonas*	Chlorophyta	α-linolenic acid (C_18__:__3_ω3)	Petrie et al., 2010
*Scenedesmus*	Chlorophyta	α-linolenic acid (C_18__:__3_ω3)	Girard et al., 2014
*Tetraselmis*	Chlorophyta	α-linolenic acid (C_18__:__3_ω3)	Kim et al., 2016
*Chlorococcum*	Chlorophyta	γ-linolenic acid (C_18__:__3_ω6)	Bhagavathy et al., 2011
*Dunaliella*	Chlorophyta	γ-linolenic acid (C_18__:__3_ω6)	Chen et al., 2015
Micromonas	Chlorophyta	Stearidonic acid (C_18__:__4_ω3)	Petrie et al., 2010
*Tetraselmis*	Chlorophyta	Stearidonic acid (C_18__:__4_ω3)	Kim et al., 2016
Porphyridium	Rhodophyta	Arachidonic acid (C_20__:__4_ω6)	Su et al., 2016
*Chlorella*	Chlorophyta	Eicosapentaenoic acid (EPA, C_20__:__5_ω3)	Kholif et al., 2017
*Dunaliella*	Chlorophyta	Eicosapentaenoic acid (EPA, C_20__:__5_ω3)	Chen et al., 2015
*Monodus*	Eustigmatophyceae	Eicosapentaenoic acid (EPA, C_20__:__5_ω3)	Chauton et al., 2015
*Nannochloropsis*	Eustigmatophyceae	Eicosapentaenoic acid (EPA, C_20__:__5_ω3)	Pal et al., 2011
*Nitzschia*	Diatom	Eicosapentaenoic acid (EPA, C_20__:__5_ω3)	Mao et al., 2020
*Odontella*	Diatom	Eicosapentaenoic acid (EPA, C_20__:__5_ω3)	Guihéneuf et al., 2010
*Pavlova*	Haptophyta	Eicosapentaenoic acid (EPA, C_20__:__5_ω3)	Guihéneuf et al., 2010
*Phaeodactylum*	Diatom	Eicosapentaenoic acid (EPA, C_20__:__5_ω3)	Cui et al., 2021
*Tetraselmis*	Chlorophyta	Eicosapentaenoic acid (EPA, C_20__:__5_ω3)	Tsai et al., 2016
*Crypthecodinium*	Dinoflagellate	Docosahexaenoic acid (DHA, C_22__:__6_ω3)	Enzing et al., 2014
*Isochrysis*	Haptophyta	Docosahexaenoic acid (DHA, C_22__:__6_ω3)	Pei et al., 2017
*Tisochrysis*	Haptophyta	Docosahexaenoic acid (DHA, C_22__:__6_ω3)	Hu et al., 2019
*Schizochytrium*	thraustochytrids^1^	Docosahexaenoic acid (DHA, C_22__:__6_ω3)	Hu et al., 2020
*Pyramimonas*	Chlorophyta	Docosahexaenoic acid (DHA, C_22__:__6_ω3)	Boelen et al., 2013

*^1^Although commonly referred as microalgae, thraustochytrids do not contain chloroplasts and are obligate heterotrophic protists.*

### Biosynthetic Pathways

Information on the biosynthetic pathways of protistan fatty acids, especially PUFAs, is crucial to define strategies for increasing their cellular abundance. Most knowledge of their pathways derives from plants, although biosynthetic mechanisms have been elucidated for thraustochytrids (Sun et al., 2019) and microalgae such as *Nannochloropsis* spp. (Vieler et al., 2012), *P. tricornutum* (Yang et al., 2013), *I. galbana* (Adarme-Vega et al., 2012), and *Chlamydomonas reinhardtii* (Giroud et al., 1988).

#### *De novo* Fatty Acid Biosynthesis

**FIGURE 1:**
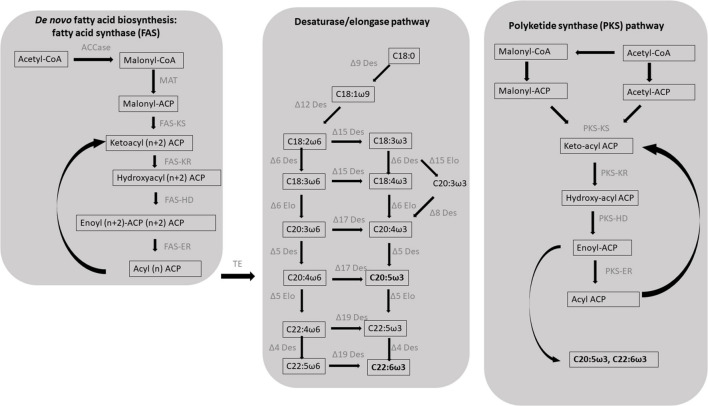
Schematic diagram of the two major biosynthetic pathways leading to the formation of the eicosapentaenoic (EPA, C_20__:__5_ω3) and docosahexaenoic (DHA, C_22__:__6_ω3) in protists. EPA and DHA in microalgae typically result from *de novo* fatty acid synthesis followed by the desaturase/elongase pathways; an alternative pathway, in which double bonds result from incomplete elongation rather than desaturation, has been identified in a class of heterotrophic heterokonts known as thraustochytrids: the polyketide synthase (PKS) pathway. ACCase, acetyl-CoA carboxylase; ACP, acyl carrier protein; MAT, malonyl-CoA ACP transacylase; KS, β-ketoacyl-ACP synthase; KR, β-ketoacyl-ACP reductase; HD, β-hydroxyacyl-ACP dehydrase; ER, enoyl-ACP reductase; TE, acyl-ACP thioesterases; Des, desaturase; Elo, elongase. Figure modified from Hu et al. (2020) and Sun et al. (2018).

*De novo* fatty acid biosynthesis starts with the condensation of an acetyl-CoA molecule with the malonyl-acyl carrier protein (ACP) by a ketoacyl synthase (KS), resulting in the formation of a β-ketoacyl intermediate with two additional carbons compared to the initial acetyl molecule (Figure 1). Subsequently, the 3-keto group is fully reduced to an alkyl group after three reactions, ultimately forming a fully saturated acyl intermediate: a ketoacyl reductase (KR) reduces the initial 3-ketoacyl-ACP intermediate to a 3-hydroxyacyl ACP, a hydroxyacyl dehydratase (HD) then converts it to enoyl-ACP which is finally saturated by an enoyl reductase (ER) (Busta and Jetter, 2018). Six to seven full elongation cycles are necessary for the biosynthesis of C_16__–__18_ fatty acids. In contrast with KR, HD and ER enzymes that can accept substrates of different length, KS enzymes are substrate-specific and three different isoforms are required for the biosynthesis of C_16__–__18_ fatty acids: KS III (C_2_ to C_4_), KS I (C_4_ to C_16_), and KSII (C_16_ to C_18_) (Millar and Kunst, 1997).

The main enzyme involved in *de novo* fatty acid biosynthesis is the fatty acid synthase (FAS) that can be classified in two types: mammals and yeasts possess the type I FAS which is a large multidomain protein whereas type II FAS typically occurs in prokaryotes and consists of four dissociable proteins carrying different catalytic activities. Since chloroplasts evolved from the endosymbiosis of a cyanobacterium by an ancestral eukaryotic cell (Harwood et al., 2017; Heil et al., 2019), type II FAS can also be found in plastid-bearing organisms such as plants and algae. Specifically, fatty acid biosynthesis in microalgae is performed by stromal type II FAS consisting in four monofunctional enzymes, each carrying a specific catalytic activity (Khozin-Goldberg, 2016). Nevertheless, putative type I FAS has been identified in *N. oceanica* and *Euglena gracilis* (Hoffmeister et al., 2005; Vieler et al., 2012).

#### Aerobic Polyunsaturated Fatty Acid Formation

After *de novo* biosynthesis, newly formed C_16__–__18_ fatty acids can undergo different rounds of elongation and desaturation leading to the formation of PUFAs. PUFAs are biosynthesized via the aerobic biosynthetic pathway in most microalgae including green algae, haptophytes, dinoflagellates, and diatoms (Jovanovic et al., 2021). The aerobic biosynthetic pathway consists in *de novo* fatty acid biosynthesis followed by different rounds of chain elongations and desaturations. The C_18_ fatty acids undergo two sequential desaturations rounds catalyzed by the Δ^9^ and Δ^12^ (or ω6) desaturases resulting in the formation of the C_18:2_ω6 intermediates which can be converted in C_18:3_ω3 by a Δ^15^ω3 desaturase. The latter products undergo a series of desaturations and elongations ultimately resulting in the formation of the ω6 derivative ARA for the C_18:2_ω6 fatty acid, and of the ω3 derivatives EPA and DHA for C_18:3_ω3 fatty acid (Figure 1; Li-Beisson et al., 2019). In some species, EPA production can be achieved by the Δ^8^ pathway that consists in an initial elongation of the C_18:3_ω3 to form C_20:3_ω3 that is then desaturated to C_20:4_ω3 by a Δ^8^ desaturase (Figure 1; Qi et al., 2002; Guschina and Harwood, 2006).

#### Anaerobic Polyunsaturated Fatty Acid Biosynthesis and Polyketide Synthases

An alternative non-oxygen dependent pathway for PUFA biosynthesis involves polyketide synthase (PKS) enzymes. Anaerobic PUFA production has been reported in bacteria as well as thraustochytrids (Figure 1; Metz et al., 2001; Meesapyodsuk and Qiu, 2016; Sun et al., 2019; Hu et al., 2020). PKSs share evolutionary similarities with FASs but they typically lack one or more catalytic sites required for a complete fatty acid elongation, leading to the formation of a longer acyl chain functionalized with either a keto group, a double bond, or a secondary alcohol group (Jenke-Kodama et al., 2005). In this case, PUFAs result from PKS enzymes that can perform some of the steps required for fatty acid elongation, without ER activity, thus forming longer products with a double bond along the aliphatic chain (Figure 1). PKS pathway for PUFAs involves fewer intermediates and consumes less NADPH compared to the desaturase/elongase pathway; for example, the formation of EPA and DHA from malonyl-CoA and acetyl-CoA, through the FAS and desaturase/elongase pathways, requires 21 and 26 NADPH units, respectively, whereas the same biosynthetic processes occurring through the PKS pathways consume 13 and 14 NADPH units, respectively (Sun et al., 2019).

### Enhancing Polyunsaturated Fatty Acid Content in Microalgae

Because of their beneficial effects on human health, the nutraceutical and livestock industries are interested in producing foods and animal feeds with enhanced ω3-PUFA content. In this context, microalgae are considered a promising source of these compounds since they are the major producers of both EPA and DHA (Sukenik, 1991; Yongmanitchai and Ward, 1991; Henderson and Mackinlay, 1992; Meireles et al., 2003b; Řezanka et al., 2010; Sayanova et al., 2011). Nevertheless, commercialization of PUFAs from microalgae is still a great challenge since several hurdles render their production economically unsustainable and not competitive with fish oil. The high operational costs are mainly due to the cultivation and downstream processing (e.g., desalting, biomass harvesting). During the last decades, intensive research has been conducted to develop different scale processing strategies for microalgal PUFA production. The starting point of the production process is the selection of PUFA-rich microalgal species (Table 1). In this context, the cultivation of *Nannochloropsis* spp. (Eustigmatophyceae) is particularly interesting for this purpose due to their high EPA content, that can account for the 35% of the total lipid composition, and for DHA absence, a feature that is particularly suitable for dietary purpose that require the production of a single specific ω3-PUFA (Khozin-Goldberg et al., 2011). High proportions of EPA are also present in other Eustigmatophyceae (Vazhappilly and Chen, 1998), diatoms such as *Amphora* sp. (Talebi et al., 2013), *P. tricornutum* (Yongmanitchai and Ward, 1991) and *Chaetoceros muelleri* (Gao et al., 2013), green algae like *C. reinhardtii* (James et al., 2011), *Chlorella minutissima* (Vazhappilly and Chen, 1998), *Dunaliella salina* (Bhosale et al., 2010), and *Scenedesmus* sp. (Talebi et al., 2013) as well as Haptophyta such as *I. galbana* (Tzovenis et al., 1997), *Pavlova lutheri* (Guihéneuf et al., 2009), and *Tisochrysis lutea* (Hu et al., 2018). In contrast, heterotrophic species such as the dinoflagellate *Crypthecodinium cohnii* (Mendes et al., 2009) and thraustochytrids can accumulate greater proportions of DHA compared to EPA (Table 1).

Interestingly, most protists can accumulate greater proportions of lipids under environmental stress conditions. For example, nitrogen deprivation revealed a successful strategy to promote lipid accumulation in *C. muelleri* (Gao et al., 2013), *D. salina* (Yuan et al., 2019), *C. reinhardtii* (James et al., 2011), *Nannochloropsis* sp. (Pal et al., 2011), *P. tricornutum* (Valenzuela et al., 2012). In addition, the EPA content was found to increase in *P. tricornutum* after a temperature shock (Jiang and Gao, 2004) and UV irradiation (Liang et al., 2006). Culturing at salinities lower than seawater values can enhance the PUFA content of marine microorganisms as found for the heterotrophic dinoflagellate *C. cohnii* (Jiang and Chen, 1999) and *Nannochloropsis* spp. (Hu and Gao, 2006; Pal et al., 2011). Short exposure to UV-C radiation also revealed successful in increasing the cellular concentration of EPA in *Nannochloropsis* sp., making it reach 30% of total fatty acids (Sharma and Schenk, 2015).

In addition to culturing manipulations, several studies proposed the application of chemical modulators as a sustainable and cost-effective strategy to promote lipid accumulation in protists. Chemical modulators can have a direct action on lipid biosynthetic pathways enhancing the availability of precursors or inhibiting the competing pathways, or can indirectly accelerate lipid pathways by increasing cell permeability or modulating oxidative stress (Sun et al., 2019). In particular, the decrease in reactive oxygen species (ROS) through the addition of antioxidants molecules was demonstrated to increase the DHA content in different microalgal species. For example, DHA accumulation likely driven by a decrease in ROS damage was observed in *C. cohnii* and *Schizochytrium* sp. (thraustochytrids) after that cultures were supplemented with sesamol and ascorbic acid, respectively (Liu et al., 2015; Ren et al., 2017).

Although the optimization of physicochemical parameters and the addition of chemical modulators can contribute to enhance PUFA production, biotechnological research aims at improving strains through genetic modifications or other manipulations in order to develop new phenotypes with a higher biotechnological potential (Ademakinwa et al., 2017). At present, the main methodologies for strain improvement include: (1) adaptive laboratory evolution (ALE), (2) random mutagenesis, and (3) genetic engineering. ALE is based on the natural selection of specific populations that acquire beneficial mutations under the pressure of prolonged stress conditions (light, pH, salinity). Such strategy is a powerful tool to yield phenotypes with interesting features such as faster growth and higher lipid content, even when genomes and metabolic pathways are unknown (Arora et al., 2020). In the context of PUFA production, ALE approach has been applied to different species and revealed promising results. Sun et al. (2018) carried out a two-factor ALE experiment based on prolonged cultivation of *Schizochytrium* sp. under culture manipulations known to increase PUFA content and to affect antioxidant production, such as low temperature and high salinity (Jahnke and White, 2003). One of the end point strains exhibited higher DHA content and lower levels of ROS species compared to parental strains coupled to an increase of gene expression levels of antioxidant enzymes and PKSs (Sun et al., 2018). A high-lipid producing *C. cohnii* strain was obtained using a chemical modulator-based ALE approach by Diao et al. (2019). Specifically, the addition of sethoxydim, an inhibitor of acetyl-CoA carboxylase, resulted in the accumulation of DHA as well as total lipids by about 90% and 50%, respectively (Diao et al., 2019).

Similarly to ALE approaches, random mutagenesis aims at generating mutants with improved features through the exposure of microalgal strains to chemical or physicals mutagens. In *Nannochloropsis* sp., treatment with the mutagen ethyl methanesulfonate promoted the formation of mutant strains with a greater fatty acid content, although a strong decrease in EPA fraction was also reported (Doan and Obbard, 2012). Conversely, exposure to N-methyl-N-nitrosourea in *N. oculata* resulted in the evolution of strains with a concomitant increase of the total fatty acid and EPA fraction (Chaturvedi et al., 2004). A substantial increase in EPA and DHA content (more than 30%) was obtained after UV-light exposure in *P. lutheri* (Meireles et al., 2003a). Similarly, the lipid productivity potential of *Scenedesmus* sp. was improved through UV mutagenesis and H_2_O_2_ treatments and an increase in lipid content by up to 55% was registered in mutagenized *Scenedesmus* sp. compared to the native strain (Sivaramakrishnan and Incharoensakdi, 2017).

The availability of microalgal genomes sequenced in the last two decades (Keeling et al., 2014; Richter et al., 2020) paved the road to genetic engineering approaches. Within this context, direct targeting of enzymes involved in PUFA biosynthesis has emerged as a promising approach to enhance the EPA and DHA content in microalgae. To date, most studies focus on a limited number of microalgal species for which the genome has been fully sequenced; these include *P. tricornutum*, *Thalassiosira pseudonana* and *N. oceanica*. The most common strategy to enhance the accumulation of ω3-PUFAs in microalgae aims at overexpressing the enzymes involved in the Δ^6^ pathway (Adarme-Vega et al., 2012). The single overexpression of endogenous Δ^5^ and Δ^6^-desaturases in *P. tricornutum* resulted in an increase in EPA content by 58% (Peng et al., 2014) and 48% (Zhu et al., 2017), respectively, along with a general increase of other PUFAs. An eightfold increase in DHA content was achieved in *P. tricornutum* through the simultaneous expression of a Δ^5^-elongase and a Δ^6^-desaturase from *Ostreococcus tauri* (Hamilton et al., 2014). Overexpression of desaturase enzymes to enhance EPA content is also promising for *N. oceanica*. Specifically, both the overproduction of a Δ^5^ or Δ^12^ desaturases led to an 25% increase in EPA per mole of total fatty acids (Poliner et al., 2018), while the overexpression of a Δ^6^ desaturase resulted in a remarkable increase of EPA reaching up to 62 mg/g of dry weight (Yang et al., 2019). In addition to desaturase enzymes, fatty acid elongases have also been targeted to increase PUFA content. For example, the overexpression of three different fatty acids elongases caused a significant increase (2.3–4.3-fold) of DHA content during the exponential growth phase (Cook and Hildebrand, 2016) in *T. pseudonana.*

### State of Art of Polyunsaturated Fatty Acid Production From Protists

Because of the fundamental role of ω3-PUFAs for the correct development and functioning of the human body, the global demand for foods and food supplements enriched with this class of fatty acids has undergone an extraordinary increase. The global market of ω3-PUFAs was worth USD 2.49 billion in 2019 with an expected annual increase of 7.7% until 2027^1^. At present, fish oils account for 79% of EPA and DHA market value while C_20__–__22_ PUFAs from microalgae cover only 18% of the market value (Voort et al., 2017). Fish oils from sustainable fisheries are very unlikely to meet the exponentially growing PUFA demand in the future, and the use of alternative PUFA sources is required. Moreover, marine pollutants such as heavy metals and hydrocarbons can accumulate in fishes as a result of biomagnification causing negative effects on human health. Even though oils containing ω3-PUFAs of microalgal origin are less competitive nowadays in terms of costs, several aspects render microalgae culturing for ω3-PUFA production promising. Among all, food and food supplements produced from microalgae are suitable for both the vegetarian and the vegan markets. To date, most of the microalgae-derived dietary supplements available on the market are obtained from whole dried cells of *Arthrospira* spp. (formerly known as *Spirulina* spp.) and *Chlorella* spp. and the producers are located mainly in Asia and United States for *Arthrospira* spp., and in Asia and Germany for *Chlorella* spp. (Enzing et al., 2014). Only in recent years, producers have been focusing on single highly valuable compounds such as DHA and EPA, that can be sold as components of dietary supplements and food ingredients. DSM-Martek Biosciences (United States) produces baby milk enriched in DHA (>30%) from *C. cohnii* as well as food supplements derived from *Schizochytrium* spp. with DHA >33 and 13.5% of docosapentaenoic acid. Lonza Group (Switzerland) produces oil capsules used as food supplement containing at least 43% DHA among the total fatty acids while the Photonz Corp. (New Zealand) has developed pharmaceutical grade EPA oil from *Nitzschia laevis* (Calado et al., 2018).

## Fatty Acid Derivatives From Microalgae, Biosynthetic Pathways and Biotechnological Applications

**FIGURE 2:**
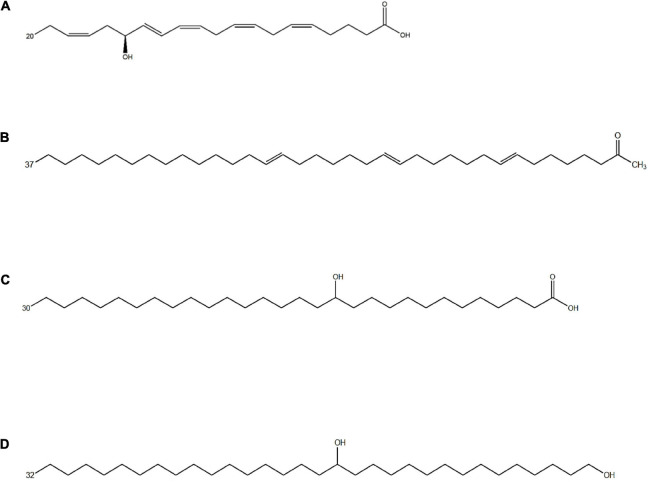
Chemical structure of different fatty acid derivatives of microalgal origin: **(A)** 15-OH-C_20__:__5_ω3 (15-HEPE), an oxylipin from *Nannochloropsis gaditana* resulting from EPA oxidation (de los Reyes et al., 2014); **(B)** C_37__:__3_ methyl alkenones from *Emiliania huxleyi* (Volkman et al., 1980); **(C)** 13-OH-C_30__:__0_ and **(D)** C_32__:__0_ 1,15 diol from *Nannochloropsis* spp., likely resulting from a PKS-catalyzed incomplete fatty acid elongation (Balzano et al., 2019).

In addition to being essential primary metabolites, fatty acids can also enter different metabolic pathways forming a wide range of secondary metabolites (Figure 2). Fatty acid derivatives possess additional points of functionalization along the alkyl chain mostly corresponding to hydroxy, keto, and epoxy functional groups, and the carboxyl end group might also undergo modifications (Millar et al., 2000).

Fatty acid derivatives have drawn great attention for a number of biotechnological applications and can make up sustainable alternatives to petroleum-based lipids (Table 2). The presence, within algal biomass, of highly reduced fatty acid derivatives can increase the overall combustion enthalpy thus improving the biofuel potential. In addition, hydroxy fatty acids can also improve the lubricant properties, and, along with other fatty acid derivatives, can be valuable substrates for several industrial applications (Mubofu, 2016) because of their additional functionalization points conferring them higher reactivity compared to non-functionalized fatty acids (Hou et al., 1999; Millar et al., 2000; Napier, 2007). Aliphatic lipids containing multiple functionalization points have, in general, drawn great attention from the chemical industry since they can be used as alternative substrates for the production of sustainable biomaterials. Finally, a number of fatty acid derivatives are biotechnologically interesting for their potential exploitation in pharmaceutical and cosmeceutical field.

**TABLE 2:** Microalgal fatty acid derivatives of biotechnological interest.

Compound class	Potential biotechnological applications	Compound	Main Microalgal producers	References
Alkenones	Biofuels	C_37__:__3_ methyl alkenone	*Gephyrocapsa huxleyi, Gephyrocapsa oceanica, Isochrysis* sp.	Bakku, 2018
	Sunscreens	C_37__:__2_ methyl alkenone	*Isochrysis* sp.	Huynh et al., 2019
	Lipsticks	C_37__:__2_ methyl alkenone	*Isochrysis* sp.	Huynh et al., 2020
	Phase-change materials (PCMs)	C_38__:__2_ ethyl alkenone	*Isochrysis* sp.	O’Neil et al., 2019
	Jet fuel	C_38__:__3_ ethyl alkenone	*Isochrysis* sp.	O’Neil et al., 2015
Hydroxy fatty acids	Lubricants	13-OH-C_30__:__0_, 15-OH-C_32__:__0_	*Nannochloropsis* sp.	Mubofu, 2016 ^1^
			*Tricleocarpa jejuensis*	Zha et al., 2020
	Skin moisturizers		*Pediastrum duplex*	Yarkent et al., 2020
			*Chlamydomonas reinhardtii*	
			*Chlorella pyrenoidosa*	
			*Cyanidium caldarium*	
Long chain diols	Biopolymer synthesis	C_30_ diols	*Nannochloropsis* sp.	Balzano et al., 2019
		C_28–30_ diols	*Proboscia* sp.	Volkman et al., 2018
		C_28–32_ diols	*Apedinella radians*	Zhang and Volkman, 2017
Oxylipins	Anti-inflammatory and anticancer drugs	13-OH-C_18:2_, 13-OH-C_18:3_	*Chlamydomonas debaryana*	Ávila-Román et al., 2016
		15-OH-C_20:5_	*Nannochloropsis gaditana*	de los Reyes et al., 2014
		2E,4E-decadienal, 2E,4E/Z,7Z-decatrienal 2E,4E-heptadienal, 2E,4E-octadienal	*Thalassiosira Rotula, Skeletonema costatum, Pseudo-nitzschia delicatissima*	Miralto et al., 1999; Cutignano et al., 2011

*^1^This study is related to castor oil that is a hydroxy fatty acid shorter than microalgal hydroxy fatty acids.*

### Oxylipins

The term oxylipins refers to a wide group of lipid metabolites deriving from the oxygenation of PUFAs. Oxylipins act as chemical signal mediators in a variety of ecological and physiological processes. They exert detrimental effects on copepod reproduction impairing the egg hatching success as well as the embryo and larval development (Poulet et al., 1995; Miralto et al., 1999; Fontana et al., 2007b). In addition to their harmful role on grazers, oxylipins can also act as chemical messengers of unfavorable conditions within phytoplankton communities regulating diatom population density through the activation of apoptosis-like processes (Ribalet et al., 2007). Oxylipins are not detectable in intact cells but they are generally released after cell damages typically caused by grazing activities (Jüttner et al., 2001).

Oxylipins encompass a broad diversity and are classified in two categories: short-chain polyunsaturated aldehydes (PUAs), and non-volatile oxylipins. PUAs are present only in few diatoms and four major compounds have been reported to date: decadienal, octadienal, octatrienal, and heptadienal (Miralto et al., 1999; d’Ippolito et al., 2005; Wichard et al., 2005). Non-volatile oxylipins occur instead in most diatom species and are defined as fatty acid derivatives with a molecular weight higher than PUAs carrying hydroperoxy-, hydroxy-, keto-, oxo-, and hydroxy-epoxy functionalities (Figure 2). Several ecological functions have been hypothesized for non-volatile oxylipins but their exact role is not fully clear. *Chaetoceros didymus* can release a series of hydroxylated EPAs that inhibit the cell growth of the lytic algicidal bacterium *Kordia algicida* allowing diatoms to survive and to dominate the phytoplankton community in the presence of algicidal bacteria (Meyer et al., 2018).

**FIGURE 3:**
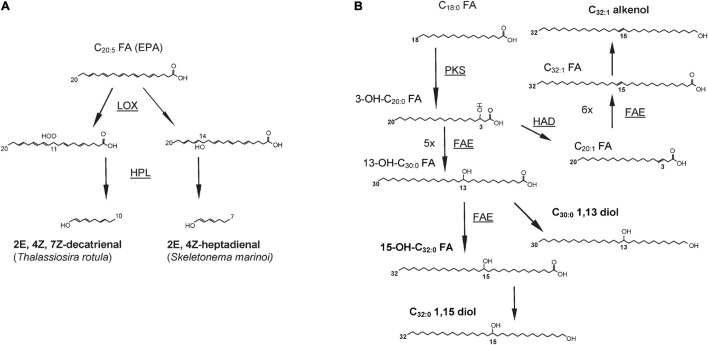
Biosynthetic pathways for **(A)** decatrienal and heptadienal, two common oxylipins produced by diatoms and **(B)** three classes of lipids produced by *Nannochloropsis* spp. and other Eustigmatophyceae: long chain hydroxy fatty acids (LCHFAs), long chain diols (LCDs), and long chain alkenols (LCAs). End products are in bold whereas enzyme names, where known, are underlined and shown next to the arrows. LOX, lipoxygenase; HPL, hydroperoxide lyase; PKS, polyketide synthase; FAE, fatty acid elongase; HAD, hydroxyacyl dehydratase. Figures redrawn from **(A)** Fontana et al. (2007a) and **(B)** Balzano et al. (2019).

Oxylipins are formed after the oxidation of one or more double bonds in PUFAs catalyzed by lipoxygenase enzymes (de los Reyes et al., 2014; Nanjappa et al., 2014). Most PUAs result from the oxidation of EPA; for example, decatrienal and heptadienal are biosynthesized after the oxidation of the EPA double bonds at the 11th and 14th positions, respectively (Figure 3) and then converted in PUAs by the hydroperoxide lyases (d’Ippolito et al., 2006). The insertion of the hydroperoxide group in different positions by the LOX enzymes and the subsequent downstream reactions contribute to the production of a broad diversity of oxylipins (Lamari et al., 2013; Nanjappa et al., 2014). The biosynthesis of oxylipins is triggered by the release of PUFAs from membrane lipids. These free fatty acids then undergo the addition of a hydroperoxide group catalyzed by lipoxygenases enzymes (LOXs) in correspondence of a double bond, resulting in the formation of fatty acid hydroperoxide intermediates (Jüttner et al., 2001; Cutignano et al., 2011).

At present, oxylipins are considered biomolecules with potential therapeutic applications due to their anticancer, anti-inflammatory, and antimicrobial properties. The first evidence on the anticancer potential of oxylipins was documented by Miralto et al. (1999). Specifically, diatom oxylipins, the 2*E*,4*E*-decadienal and the 2E,4E/Z,7Z-decatrienal exhibit antiproliferative and apoptotic activities in human colon adenocarcinoma cell lines Caco-2 (Miralto et al., 1999). In addition to anticancer properties, several studies reported evidence of the anti-inflammatory beneficial effects of different oxylipins. A series of hydroxy C_16_ and C_18_ PUFAs isolated from *Chlamydomonas debaryana* and a C_20_ hydroxy acid from *Nannochloropsis gaditana* were shown to inhibit the production of the potent pro-inflammatory cytokine tumor necrosis factor α (TNF-α) which is released by monocytes and macrophages during inflammatory processes (de los Reyes et al., 2014). Similarly, oral administration of oxylipin-rich biomass from *C. debaryana* exhibited anti-inflammatory properties in an induced murine recurrent colitis model. It was reported a decrease in pro-inflammatory cytokines TNF-α, the interleukins (ILs) IL-1β, IL-6, and IL-17, a decrease in the level of inducible nitric oxide synthase (iNOS), cyclooxygenase 2 (COX-2) and NF-κB, as well as an increase of the anti-inflammatory transcription factor PPAR-γ (Ávila-Román et al., 2016). Subsequently, Ávila-Román et al. (2018) proposed a molecular mechanisms responsible for the anti-inflammatory effects of the major oxylipins produced by *C. debaryana* and by *N. gaditana*. First, the 13-OH-C_18__:__3_, and the 13-OH-C_18__:__2_, from *C. debaryana* as well as the 15-OH-C_20__:__5_ from *N. gaditana* enhance PPAR-γ nuclear translocation. In parallel, they also lower the activation of the transcription factor NFκB, thus resulting both in the inhibition of the transcription inflammatory genes iNOS and COX-2, and in a decrease in the production of the inflammatory molecules TNF-α, IL-1β, IL-6, IL-8 (Ávila-Román et al., 2018).

### Alkenones

Another group of fatty acid derivatives of microalgal origin are the alkenones, that are polyunsaturated C_36–40_ ketones possessing 2 to 4 *trans* double bounds and a methyl or ethyl keto group in terminal position (Volkman et al., 1980; Marlowe et al., 1984). To date, these unique lipids have been detected in four genera of Haptophyta: *Gephyrocapsa*, *Isochrysis*, *Tisochrysis*, and *Chrysotila* (Volkman et al., 1980; Marlowe et al., 1984). Different environmental factors such as salinity, temperature, and nutrient availability influence the composition and the degree of unsaturation. C_37_ and C_39_ alkenones (Figure 2) with two or three double bonds are the most abundant alkenones in marine and lacustrine environments and are mainly produced by *Gephyrocapsa huxleyi* (formerly known as *Emiliania huxleyi*) and *Gephyrocapsa oceanica* (Volkman et al., 1980; Cranwell, 1985; Conte et al., 1995; Sawada et al., 1996). The cellular concentration of alkenones has been found to increase during the stationary phase of algal growth (Epstein et al., 2001; Eltgroth et al., 2005), under nitrogen deprivation (Bakku, 2018), and to decrease under prolonged darkness (Epstein et al., 2001; Eltgroth et al., 2005), suggesting that alkenones play a role as storage lipids. In agreement with the hypothesis, several studies demonstrated that alkenones are stored inside lipid bodies in *T. lutea, I. galbana*, and *G. huxleyi* (Eltgroth et al., 2005; Shi et al., 2015). The ratios among different alkenones in the sediment have been shown to reflect ancient seawater temperatures (Müller and Fischer, 2004), hence the dynamics of these lipids have been investigated by a range of geochemical studies (Volkman et al., 2018).

Little is known about the biosynthesis of alkenones. Since alkenone concentration was found to increase under nitrogen-deprivation (Eltgroth et al., 2005; Tsuji et al., 2015; Weiss et al., 2019), and their degree of unsaturation varies at changing temperatures (Araie et al., 2018) analogously to fatty acids, they have been suggested to be produced in the chloroplasts and to be formed from fatty acids. Since alkenones attract also biotechnological interest, current research is focusing on delineating the biosynthetic pathways of such compounds and to develop genetically engineered strains with increased alkenone concentrations^2^. Alkenones were tested as sustainable wax components alternative to commercially available waxes for a series of cosmeceutical and personal care products. The addition of alkenones to sunscreens has been shown to improve protection from sunlight-associated UVs without increasing the apparent viscosity of the sunscreen, thus exhibiting a performance similar to that of commercially available waxes (Huynh et al., 2019). In addition, alkenones were also evaluated as structuring agents for lipsticks. Lipsticks with the highest alkenone content (7%) exhibited the most desirable attributes including ease of bending, high level of firmness, low pay-off in terms of amount, high color intensity on skin and low friction (Huynh et al., 2020).

Another potential application of alkenones relates to phase-change materials (PCMs). PCMs are substances that are able to absorb and release large quantities of energy while they undergo a sharp temperature change. They are commonly used for thermal insulation in commercial applications in which stable temperature and/or energy storage conditions are required; PCMs are typically manufactured using petroleum-derived paraffin waxes. Alkenones possess thermal properties that are relevant for the development of renewable PCMs (O’Neil et al., 2019). O’Neil et al. (2019) evaluated the thermal properties of alkenones isolated from *Isochrysis* sp. and found a greater thermal stability and a similar latent heat of fusion compared to commercial PCMs.

Alkenones are also considered as lipids suitable for biofuel development. Although alkenones cannot be used directly for biofuel production because of their high boiling points (>60°C) compared to TAGs, the possible advantages of alkenones include the greater stability against photo oxidation due to the embedded *trans* double bond geometry (Rontani et al., 2006) and the absence of the glycerol backbone (O’Neil et al., 2014, 2015, 2019). Several attempts have been made to convert alkenones in potential substrates suitable for the production of renewable liquid fuels, these include the pyrolysis or butenolysis, methodologies that lead to the formation of short chain hydrocarbons like n-alkanes and jet-fuels (Wu et al., 1999; Bakku, 2018; O’Neil et al., 2019).

### Waxes

Waxes are attracting the interest of the biotechnological industry because of their high resistance to mechanical damages and chemical degradation. They have been mostly studied in the plant cuticle, a lipid layer that coats aerial organs providing protection from desiccation.

Plant cuticle contains a biopolymer, known as cutin, and a mixture of different waxes. Cutin consists of monomers of ω- and mid-chain hydroxy and epoxy C_16_ and C_18_ fatty acids cross-linked by ester bonds (Nawrath, 2002). Cuticular waxes are a complex mixture of very long chain fatty acid (C_20_-C_34_) derivatives that can be either ubiquitous or taxa-specific. Ubiquitous waxes consist in fully saturated very long chain alcohols, aldehydes, and fatty acids (Jetter et al., 2007). Dimers of fatty acids and wax alcohols linked through ester bonds have also been found (Samuels et al., 2008). Taxa-specific waxes instead consist of fully saturated linear aliphatic chains with 29 or 31 carbons that may contain two alcohol or keto groups. In taxa-specific waxes, the presence of one or more functional groups gives rise to a greater structural diversity (Jetter et al., 2007). In addition to plants, wax esters have been observed also in mammals (Cheng and Russell, 2004), birds (Hellenbrand et al., 2011), and insects (Teerawanichpan et al., 2010; Jaspers et al., 2014; Tupec et al., 2017).

Within microalgae, wax esters have been found in the genus *Euglena*. *Euglena* spp. can convert, under anaerobic conditions, the storage polysaccharide paramylon to wax esters through a unique mechanism known as wax ester fermentation (Teerawanichpan and Qiu, 2010; Inui et al., 2017). The resulting wax esters consist of a series of fully saturated C_10__–__18_ fatty acids and alcohols, the most dominants being the C_14__:__0_ fatty acid (myristic acid) and C_14__:__0_ fatty alcohol (myristyl alcohol), that account for 44% and 47% of the fatty acid and alcohol moieties of the wax esters, respectively (Inui et al., 1983). Wax esters have also been suggested to occur in the cell walls of some green algae. Cell wall lipids of *Neochloris oleoabundans* mostly consist in palmitic and stearic acids as well as fatty acids with >20 carbons (Rashidi and Trindade, 2018), that are typically considered as the precursors in the elongation pathways of cutins and waxes (de Leeuw et al., 2006).

The biosynthesis of wax esters has been extensively investigated in plants (Kunst and Samuels, 2009) as well as in the honey bee *Apis mellifera* (Blomquist et al., 1980), but little is known on microalgae. Even-numbered fatty acyl-CoAs possessing up to 38 carbons can be formed, serving as precursors for wax biosynthesis (Busta and Jetter, 2017). Analogous to *de novo* fatty acid biosynthesis, the FAE complex catalyzes the formation of VLCFAs through four enzymatic reactions leading to the addition of two carbon units to the growing acyl-CoA chain. While carbon units are added as malonyl-ACP to the elongating acyl chain in FAS-based pathways, FAE enzymes use malonyl-CoA as carbon donor (Samuels et al., 2008).

For the biosynthesis of wax components, fatty acids can undergo head group modifications through two different processes: the decarbonylation and the acyl reduction pathways (Kunst and Samuels, 2003; Samuels et al., 2008). The decarbonylation pathway is a two-step process in which fatty acid reduction leads to the formation of even-numbered aldehydes that, in the second step, the carbonyl group is cleaved generating odd-chain alkanes (Bernard et al., 2012). Alkanes can be eventually hydroxylated to form odd-numbered secondary alcohols and the hydroxy group can be further oxidized to form ketones. The acyl reduction pathway consists in the reduction of the carboxylic group to an alcohol group catalyzed by the fatty acyl-CoA reductase (FAR) followed by the a reaction between the resulting alcohols and a fatty acyl-CoA catalyzed by a wax ester synthase (WS) to form alkyl esters (Li et al., 2008). Similarly to multicellular organisms, the reduction of fatty acids to alcohols by a FAR, followed by the reaction of the fatty alkyl ester formation catalyzed by a WS, has been documented in microalgae such as *E. gracilis* (Teerawanichpan and Qiu, 2010) and *P. tricornutum* (Cui et al., 2018). Based on similarities with the WS from *P. tricornutum*, genes coding for WS enzymes have been also predicted in the genomes of other heterokonts (*Aureococcus anophagefferens*, *N. gaditana*) as well as green algae from the genera *Micromonas*, *Ostreococcus*, and *Bathycoccus* (Cui et al., 2018) and the species *N. oleoabundans* (Rashidi and Trindade, 2018) and *Klebsormidium flaccidum* (Kondo et al., 2016). In contrast, the WS from *E. gracilis* differs significantly from that of *P. tricornutum* exhibiting similarities with bacterial WSs (Tomiyama et al., 2017). Current data suggest that at least two independent pathways for WS biosynthesis evolved in microalgae and wax esters are likely to be present in several species.

Wax esters from *Euglena* are interesting for biofuel production. In particular, the major constituents of *Euglena* wax esters, the myristic acid and myristyl alcohol, are particularly suitable as precursors for the synthesis of drop-in jet fuel since they possess a lower freezing point/high cetane number compared to C_16–18_ fatty acids (Klopfenstein, 1985; Inui et al., 2017).

### Long Chain Hydroxy Fatty Acids (LCHFAs), Long Chain Diols (LCDs), and Long Chain Alkenols (LCAs)

Different microalgal taxa, especially from the class Eustigmatophyceae, possess C_24__–__34_ fatty acid derivatives that are functionalized at a mid-chain position. These compounds might reveal suitable biofuel candidates as well as potential starters for the industrial synthesis of polymers.

#### Occurrence in Microalgae

Eustigmatophyceae and, to a lesser extent, other heterokonts, possess bifunctional C_28__–__32_ aliphatic lipids functionalized at the end of the chain as well as at a mid-chain position (Volkman et al., 1999a; Rampen et al., 2014a; Villanueva et al., 2014). Gelin et al. (1997b) identified a series of C_28__–__34_ hydroxy fatty acids with the hydroxy group in ω-18, and two dihydroxy fatty acids, such as 15,16-(OH)_2_-C_32_ and 16,17-(OH)_2_-C_33_ in the eustigmatophycean genus *Nannochloropsis*. The most abundant long chain hydroxy fatty acids (LCHFAs) in *Nannochloropsis* spp. are the 13-OH-C_30__:__0_ (Figure 2) and the 15-OH-C_32__:__0_ fatty acids (Gelin et al., 1997b; Balzano et al., 2017). In addition, Eustigmatophyceae produce two classes of compounds similar to LCHFAs in terms of length and position of functionalization: the long chain alkenols (LCAs) and the long chain diols (LCDs) (Volkman et al., 1992, 1999a,b). The secondary functional group, that is a hydroxy for LCHFAs and LCDs, and a double bond for LCAs, occurs, in most cases, on the 13th or 15th position for compounds with 30 carbons, and on the 15th position for the C_32_ compounds. C_32_ diols are the most abundant LCDs in *Nannochloropsis* spp. (Figure 2) whereas longer LCDs are present at lower concentrations. Minor amounts of diols with an odd number of carbons are also present in *Nannochloropsis* spp. and are dominated by ω17 isomers (Volkman et al., 1992; Gelin et al., 1997b; Mejanelle et al., 2003; Rampen et al., 2014a).

Long chain diols are also produced, along with structurally similar hydroxy alkanoates, by diatoms from the genus *Proboscia*. Sinninghe Damsté et al. (2003) found saturated and monounsaturated C_28_ and C_30_ 1,14-diols as well as 12-OH-C_27_ and the 12-OH-C_29_ methyl alkanoates in the: *Proboscia indica* and *Proboscia alata*. Diols and alkanoates were suggested to have a common origin, deriving from the 12-OH-C_26_ and the 12-OH-C_28_ fatty acids (Sinninghe Damsté et al., 2003; Rampen et al., 2007). Although LCDs have not been detected in other diatoms, as revealed by a screening on 120 strains (Sinninghe Damste et al., 2004), few species from other classes of heterokonts, such as Dictyochophyceae (*Apedinella radians* and *Florenciella parvula*), Raphidophyceae (*Heterosigma akashiwo* and *Haramonas dimorpha*), Chrysophyceae (*Chrysosphaera parvula*), and Pelagophyceae (*Sarcinochrysis marina*) have been found to contain LCDs (Rampen et al., 2011; Balzano et al., 2018). This suggests that all the heterokonts might have evolved the ability to biosynthesise LCDs and LCHFAs and that such ability has been lost in most cases. In addition to heterokonts, green and red algae have been occasionally shown to contain hydroxy fatty acids. Specifically 3-hydroxy fatty acids were found in *Pediastrum duplex, C. reinhardtii*, *Chlorella pyrenoidosa*, and *Cyanidium caldarium* (Matsumoto and Nagashima, 1984; Yarkent et al., 2020) and mid-chain functionalized mono- and di-C_22__–__26_ hydroxy fatty acids were detected in the zygospores of *Chlamydomonas monoica* (Blokker et al., 1999). The red seaweed *Tricleocarpa jejuensis* contains mid-chain hydroxy C_18__:__1_ with algicidal properties (Zha et al., 2020) whereas ω-hydroxy fatty acids have been found in *Chlorella emersonii*, *Tetraedron minimum*, and *Scenedesmus communis* (Allard et al., 2002). ω- and mid-chain C_30__–__34_ hydroxy fatty acids have been identified in *T. minimum*, *S. communis*, and *Pediastrum boryanum* (Blokker et al., 1998). The presence of mid-chain hydroxylated fatty acids and diols in phylogenetically distant taxa such as heterokonts and Archaeplastida, suggests that either the ability to biosynthesise diols resulted from convergent evolution or many other taxa are likely to be able to biosynthesise these compounds.

The ability of other phytoplankters to biosynthesise these compounds is also suggested by the widespread occurrence of LCDs in sediments and suspended particulate matter of aquatic environments (Sinninghe Damsté et al., 2003; Rampen et al., 2007, Rampen et al., 2012, 2014b; Lattaud et al., 2017). In particular, the major marine biological sources are still unclear; while the most abundant LCD in the environment is the C_30__:__0_ 1,15 diol, the two main diols produced by *Nannochloropsis* spp., C_32__:__0_ and C_32__:__1_ 1,15 diols (Volkman et al., 1992; Rampen et al., 2014a), are absent or present at low concentrations in marine suspended particulate matter and marine sediment (Rampen et al., 2007; de Bar et al., 2016; Lattaud et al., 2017). Furthermore, the contribution of Eustigmatophyceae, *Proboscia* spp. as well as other known LCD-producers to marine microbial communities is negligible (de Vargas et al., 2015; Tragin et al., 2018) suggesting that most LCDs in the marine environment result from debris that derive, in turn, from other species of marine or freshwater origin (Balzano et al., 2018). The scattered occurrence of LCDs within heterokonts, along with their widespread presence in the marine environment, suggest that one or more unknown LCD producers are likely to account for a significant portion of the marine microalgal biomass.

#### Biosynthesis of Long Chain Diols, Long Chain Alkenols, and Long Chain Hydroxy Fatty Acids in *Nannochloropsis* spp.

LCHFAs from *Nannochloropsis* spp. are unlikely to result from in-chain hydroxylation since the secondary hydroxy groups occur at a constant ω-18 distance from the methyl end (Gelin et al., 1997b) and their cellular abundance has been found to be significantly correlated with that of C_14__–__18_ fatty acids, in laboratory cultures (Balzano et al., 2017). Furthermore, structural similarities in carbon chain length and position of the functional groups indicate a common biosynthetic pathway for LCHFAs and LCDs in *Nannochloropsis* spp. (Gelin et al., 1997a).

The combination of stable isotope labeling with culturing experiments followed by transcriptome analyses in *N. oceanica* and *N. gaditana* highlighted that C_18_, and to a lesser extent the C_16_ fatty acids, are likely to undergo an incomplete fatty acid elongation cycle leading to the formation of a 3-OH C_18__–__20_ fatty acids (Balzano et al., 2019). This reaction is likely to be catalyzed by a PKS enzyme that possesses both the KS and the KR domains but lacks the HD and ER domains. Subsequently, the resulting 3-OH-C_18__–__20_ fatty acids can undergo five or six full elongation cycles, potentially catalyzed by the FAE enzymes, to form the 13-OH-C_30__:__0_ and 15-OH-C_32__:__0_ fatty acids (Figure 3), that are the two major LCHFAs in *Nannochloropsis* spp. (Balzano et al., 2019). The carboxylic group of LCHFAs is then likely to be reduced to alcohol to form LCDs, whereas the formation of LCAs would involve the dehydration of the intermediate hydroxy group occurring either before or after the reduction of the terminal carboxylic group (Balzano et al., 2019). Similarly to *N. oceanica* and *N. gaditana*, PKS enzymes possessing only the KS and KR domains are also present in *P. alata*, and might be involved in the biosynthesis of diatomaceous LCDs (Balzano et al., 2019).

#### Long Chain Diols and Long Chain Alkenols as Cell Wall Building Blocks

LCDs and LCAs are refractory to degradation and can persist in the environment for long periods (Rodrigo-Gamiz et al., 2016). In addition, both LCAs and LCDs are thought to be strongly bound between each other and with other lipids forming the algaenans, that are acid and base-resistant aliphatic polymers present in Eustigmatophyceae (Gelin et al., 1996, 1999; Scholz et al., 2014; Zhang and Volkman, 2017) as well as in some green algae (Blokker et al., 1999; Allard et al., 2002; Kodner et al., 2009). Algaenans are thought to share chemical similarities with sporopollenin, a polymer making the outer layer of pollen. Fourier transform infrared spectroscopy analysis on *N. gaditana* cell walls revealed that LCDs and LCAs are mainly bound via ether cross-links. Minor amount of C=O stretches were also detected in algaenans and are likely to correspond to keto, ester or carboxylic functional groups of long-chain keto-ols, LCHFAs, and dihydroxy fatty acids (Scholz et al., 2014). Stepwise pyrolysis of *N. oculata* biomass confirmed that algaenans are likely to consist mostly in ether-bound LCDs (Zhang and Volkman, 2017). Fatty acid derivatives from green algae are also thought to be the building blocks of cell wall polymers. A structure similar to that of *Nannochloropsis* algaenans, but with greater proportions of ester-bound hydroxy fatty acids, has been suggested for the algaenans of green algae such as *C. monoica*, *T. minimum*, *S. communis*, and *Pediastrum boryanum* (Blokker et al., 1998, 1999). In contrast, algaenans from *Botryococcus braunii* mostly consist of aliphatic aldehydes and unsaturated hydrocarbons (Simpson et al., 2003). The outer layer of *Chlorella protothecoides* cell wall was also found to contain algaenans (He et al., 2016).

Since lipid extraction techniques mostly used to analyze fatty acid derivatives usually cleave ester linkages but are ineffective toward ether-bound lipids, the cellular abundance of eustigmatophycean LCDs, that are thought to be mostly ether-bound, are likely to be significantly higher than those typically measured. The incubation of *N. oculata* biomass in the dark, under aerobic conditions, resulted in a sharp increase of LCDs after 100 days, confirming that LCDs can derive from algaenan degradation (Reiche et al., 2018). The chemical structure of algaenans still requires to be fully elucidated, but their high stability against chemical and biological degradation makes these compounds promising for the development of sustainable materials.

#### Biotechnological Applications

Hydroxy fatty acids exhibit an interesting biotechnological potential for several applications. At present, the only commercial sources of hydroxy fatty acids are plant-derived castor and lesquerella oils, that are rich in 12-OH-C_18__:__1_ω9 (ricinoleic acid, RA) and 14-OH-C_20__:__1_ω9 (lesquerolic acid, LA), respectively. Both castor and lesquerella oils have been investigated for their biofuel potential (Berman et al., 2011; Knothe et al., 2012) revealing as potential alternatives to petroleum-based fuels. Fatty acid derivatives such as alkenones, LCHFAs, LCAs, and LCDs are longer than C_16__–__20_ fatty acids and have a larger proportion of methylene groups over the total number of carbons, resulting in a greater combustion enthalpy (Balzano et al., 2019). In addition, the functionalized carbons in LCAs and LCDs are more reduced than the carboxylic groups of fatty acids, and thus contain more energy. This suggests a great biofuel potential for microalgal biomass enriched in both TAGs and fatty acid derivatives. In particular, since *Nannochloropsis* spp. are already considered as suitable biofuel candidates, enhancing their content in LCDs, LCAs, and LCHFAs can lead to an increase in the combustion enthalpy of the resulting biomass.

In addition, methyl esters of castor and lesquerella oils were indeed found to be more efficient as lubricants compared to methyl esters from commercially available vegetable (rapeseed and soybean) oils, when added to reference fuels at low concentrations (<1.0%) (Goodrum and Geller, 2005). In addition to castor and lesquerella oil, recent studies evaluated the lubricant properties of *Orychophragmus violaceus* seed oil, which is rich in 7,18-(OH)_2_-C_24__:__1_ω9 (nebraskanic) and the 7,18-(OH)_2_-C_24__:__2_ω3ω9 (wuhanic) acids. Experiments of friction reduction and wear of sliding steel surfaces demonstrated a friction decrease by 20%, lower wear and higher temperature stability for *O. violaceus* oil over castor oil (Li et al., 2018). Similarly to hydroxy fatty acids from plants, the presence of small amounts of LCHFAs, within lipid-enriched *Nannochloropsis* biomass is likely to improve the lubricity of the resulting biofuel. The presence of small amounts of LCHFAs, potentially increasing the combustion enthalpy and improving the lubricant properties of the transesterified algal biomass, is likely to make *Nannochloropsis* spp. one of the most suitable candidates for biofuel development in the coming years.

Fatty acid derivatives with secondary functional groups might also reveal as suitable starters for the industrial synthesis of biopolymers, cosmetics, and additives in coatings and paintings. Most research on sustainable materials from fatty acid derivatives focuses on plant lipids and very little research has been carried out on microalgae. Currently RA and LA are the compounds mostly investigated by the biotechnological industry for this purpose. They have been directly tested as fuel lubricants (Goodrum and Geller, 2005) as well as starters for the synthesis of different kinds of lubricants (Cermak et al., 2013). Estolides are long chain esters deriving from hydroxy acids (Yoshida et al., 1997; Cermak et al., 2013), and estolides of both RA and LA possess striking properties as lubricants such as extremely low pour point (−54°C) and high flash point (>300°C), revealing advantageous alternatives to commercial oils (Cermak et al., 2006; Salimon et al., 2011).

Hydroxy fatty acids are also highly suitable for the chemical modifications required during the industrial synthesis of polyester and polyurethane. RA has been tested for the synthesis of segmented polyurethanes, a class of copolymers consisting of alternating hard segments, that provide rigidity and strength through cross-links, and soft segments that confer elasticity. Soft segments are usually prepared from polyethers or polyesters while hard segments consist in a diisocyanate and a chain extender, typically a short diol (Petrovic et al., 1991, 2010; Sharmin and Zafar, 2012). Soft components can be synthetized from polycondensation of two RA molecules through ester link between the terminal hydroxy groups, resulting in the formation of the polyester diols (Xu et al., 2008; Miao et al., 2014). Polyricinoleic acid is an estolide of RA of natural or synthetic origin (Bodalo-Santoyo et al., 2005). Petrovic et al. (2010) tested the biodegradability of a segmented polyurethane consisting in soft segments of polyricinoleic acid at different concentrations (40 to 70%), diphenyl methane diisocyanate and butane diol hard segments. The study revealed that polyurethanes based on polyricinoleic acid degrade faster than the corresponding petrochemical polyurethanes and that the degradation rate of such polyurethanes increases at increasing proportions of polyricinoleic acid (Petrovic et al., 2010). Moreover, RA can react also with different organic molecules to form other polymers with biotechnological properties (Miao et al., 2014). For example, the incorporation of RA into polylactic acid results in the formation of a polymer with improved pliability, hydrophobicity and softness (Slivniak and Domb, 2005). RA encounters applications also in Biomedical Sciences. For example it has been used, in combination with decanedioic acid (sebacic acid), to form the poly(ricinoleic acid-co-sebacic acid), a polymer that can be potentially used for drug delivery (Slivniak and Domb, 2005).

RA and LA can be thus used as precursors for a number of synthetic processes. Within this context, LCHFAs from microalgae are structurally similar to RA and LA, suggesting their possible use as starters for the industrial synthesis of biomaterials. The greater biomass productivity of microalgae compared to terrestrial plants suggests that the industrial production of LCHFAs might reveal advantageous. Although LCHFAs typically account for a tiny proportion of microalgal lipids and their aliphatic chain can be longer than those of RA and LA, potentially differing in their chemical behavior, genetic manipulations of the enzymes coding for LCHFAs might lead to the biosynthesis of shorter products as well as enhanced LCHFA yield within algal biomass. Because of its biotechnological potential, a full understanding of the biosynthetic mechanisms of LCHFAs is highly desirable.

### State of Art of Microalgal Fatty Acid Derivatives

Although fatty acid derivatives from microalgae encounter little applications compared to aliphatic lipids from plants (e.g., RA, waxes) to date, the extraordinary diversity of microalgae makes their metabolites at least as much promising as plant products for the production of environmentally sustainable fuels, biomaterials, as well as pharmaceutical, nutraceutical, and cosmeceutical products. In contrast with saturated and unsaturated fatty acids, microalgae typically exhibit a very poor content in fatty acid derivatives. For example, the LCD content in *Nannochloropsis* spp. can be up to two orders of magnitude lower than that of EPA (Reiche et al., 2018) or monounsaturated and saturated fatty acids (Balzano et al., 2017). The production of substantial amounts of fatty acid derivatives in microalgae is thus more time consuming and risks being economically unviable. Culturing strategies aimed at increasing the microalgal content in fatty acid derivatives (Table 3) can contribute yielding larger proportions of specific products but might still reveal insufficient for a viable exploitation.

**TABLE 3:** Strategies that can potentially increase the content of fatty acid derivatives in microalgal cultures.

Compound	Species	Culturing manipulations	References
Alkenones	*Gephyrocapsa huxleyi*, *Isochrysis galbana*	Nitrogen deprivation	Epstein et al., 2001; Eltgroth et al., 2005
Hydroxy fatty acids	*Nannochloropsis oceanica*	High light intensity	Balzano et al., 2017
Diols	*Nannochloropsis oceanica*	Prolonged darkness	Balzano et al., 2019
Oxylipins	Mixed diatom biofilm	Zooplankton grazing	Jüttner et al., 2001
Waxes	*Euglena gracilis*	Anaerobic conditions	Inui et al., 2017

To date, large scale production of microalgae is still limited by several challenges that make massive culturing economically unviable. First, only a tiny fraction of microalgae, within a culture, are directly exposed to sunlight or artificial light, while they generate a shading effect toward the other cells of the culture. In addition, strong light exposure generates photooxidation, this lowering carbon fixation rates. It has been shown that photosynthetic efficiency is ≤1.2% in open raceway ponds and lower in photobioreactors and other culturing systems, such that most of the sunlight hitting the cultures is not used for photosynthesis but rather lost as heat (Grobbelaar, 2012). In addition, a more severe limitation to microalgal mass culturing is associated with harvesting costs (Khan et al., 2018). Microalgae can be collected from liquid cultures by filtration, flocculation, or centrifugation and any of these three techniques implies significant costs when applied to large scale cultures. In spite of its great potential for mass culturing, the small size (3–4 μm) and the presence of lipid droplet leading to a lower density, make harvesting of *Nannochloropsis* spp. even more complicate compared to other microalgae (Chua and Schenk, 2017). Finally, compounds of interest are to be extracted from the microalgal biomass and the extraction process might reveal expensive, time-consuming, and require the use of toxic chemicals. The extraction of fatty acids and fatty acid derivatives from *Nannochloropsis* spp. is complicated by the presence of a rigid cell wall. The presence of an inner cellulose layer and an outer algaenan layer makes *Nannochloropsis* cells harder to lyse compared to diatoms or green algae (Chen et al., 2016). Overall, in spite of the promising features of microalgal mass culturing, several issues are to be solved to make large scale cultivation economically viable and environmentally safe. To date, with the exception of very highly valuable compounds, for which even the production of tiny amounts can reveal economically feasible, the use of microalgal products for biotechnological applications requires that the cellular concentrations of such products are further enhanced. Genetic manipulations aimed at modifying the enzymatic machinery that drives specific pathways in order to enhance the rate of biosynthesis, might contribute increasing the cellular concentration of fatty acid derivatives.

## Concluding Remarks

Increasing concerns on both environmental pollution and limited availability of fossil fuels and raw materials contributed to shift the attention of the biotechnological industry towards the development of biofuels. Fatty acid derivatives such as alkenones, LCHFAs, LCAs, and LCDs are longer than C_14__–__20_ fatty acids and contain larger proportions of methylene groups over the total number of carbons, resulting in a higher combustion enthalpy (Balzano et al., 2019). In addition, the functionalized carbons in LCAs, LCDs, and alkenones are more reduced, and thus contain more energy, compared to the carboxylic groups of fatty acids. This suggests a great biofuel potential for microalgal biomass enriched in both TAGs and fatty acid derivatives. In addition, fatty acid derivatives from microalgae can potentially encounter a number of applications such as fuel additives and starters for the industrial synthesis of different polymers.

Because of the relative easiness of culturing microalgae, large scale culturing for the production of microalgal-derived compounds can be a concrete alternative to traditional plants products. The production of microalgal specialty compounds such as fatty acid derivatives at industrial scale is limited by the low proportions of such compounds over the total microalgal biomass. Appropriate culturing manipulations may yield microalgal biomass enriched in specialty compounds, but large scale production might still reveal unviable. Genetic engineering coupled with culturing manipulations can lead to higher proportions of specialty compounds. Moreover, massive culturing needs to focus on multiple products, and the bulk biomass remaining after the extraction of specialty compounds can also to be exploited for other purposes. For example, the extraction of both fatty acid derivatives and PUFAs from microalgal biomass, combined with the exploitation of the unextracted residue for biofuel production might reveal suitable for scale-up.

The use of microalgae for the production of biotechnologically relevant compounds can also be coupled with their bioremediation potential (Mata et al., 2010). Microalgae have been successfully used for the removal of nutrients (Lei et al., 2018; Bellucci et al., 2020) and heavy metals (Kumar et al., 2015) from contaminated waters and such processes can be coupled with the production of specialty compounds such as fatty acid derivatives. In this context, several studies coupled contaminant removal from polluted waters with biomass production for biodiesel development. *Chlorella kessleri* and *Chlorella vulgaris* cultivated in urban wastewaters exhibited an efficient removal of nitrogen and phosphorus (>95%) and the resulting biomass was found to be a suitable starter for the production of both biodiesel and methane after transesterification, and anaerobic digestion, respectively (Caporgno et al., 2015). Patidar et al. (2015) investigated the biodiesel production and metal accumulation in naturally floating microalgae collected from an eutrophic lagoon during different seasons. Highest heavy metal removal occurred in the pre-monsoon season and the obtained biodiesel exhibited properties that met the European biodiesel standards. Current studies based on combining pollutant removal with the production of specialty compounds are thus promising for the development of industrial processes economically viable and environmentally sustainable.

## Author Contributions

The present review was conceived by both authors as part of MB Ph.D. program. MB drafted the manuscript and SB revised it critically. Both authors read and agreed with the final version of the manuscript.

## Conflict of Interest

The authors declare that the research was conducted in the absence of any commercial or financial relationships that could be construed as a potential conflict of interest.

## Publisher’s Note

All claims expressed in this article are solely those of the authors and do not necessarily represent those of their affiliated organizations, or those of the publisher, the editors and the reviewers. Any product that may be evaluated in this article, or claim that may be made by its manufacturer, is not guaranteed or endorsed by the publisher.

## Footnotes

1
https://www.grandviewresearch.com/industry-analysis/omega-3-market

2
http://plmet.biol.tsukuba.ac.jp/crest-en.html
